# Autologous Platelet Concentrates in the Management of Medication-Related Osteonecrosis of the Jaw: A Systematic Review

**DOI:** 10.3390/medicina61081496

**Published:** 2025-08-21

**Authors:** Filipa Ferreira, Carlos Faria, Daniel Humberto Pozza

**Affiliations:** 1 Experimental Biology Unit, Department of Biomedicine, Faculty of Medicine of Porto, University of Porto, 4200-319 Porto, Portugal; filipalsf00@gmail.com; 2 Department of Surgery and Physiology, Faculty of Medicine of Porto, University of Porto, 4200-319 Porto, Portugal; carlosafaria@gmail.com; 3 Institute for Research and Innovation in Health-i3S, University of Porto, 4200-135 Porto, Portugal

**Keywords:** platelet rich fibrin, platelet-rich plasma, osteonecrosis, bone healing, bone regeneration, regenerative medicine, bisphosphonate-related osteonecrosis, mediation-related osteonecrosis of the jaw, autologous platelet concentrates

## Abstract

*Background and Objectives*: Medication-related osteonecrosis of the jaw (MRONJ) is a challenging condition linked to antiresorptive and antiangiogenic medications. Their complex pathophysiology and resistance to standard treatments have led researchers to explore adjunctive therapies. This systematic review evaluated the effectiveness of autologous platelet concentrates—namely platelet-rich plasma (PRP) and platelet-rich fibrin (PRF)—in promoting healing, bone regeneration, and symptom relief in MRONJ patients. *Materials and Methods*: A systematic literature search was conducted using PubMed, Scopus, and Web of Science for studies that assessed the use of PRP or PRF in MRONJ management. The risk of bias and study quality were evaluated using ROB-2 and ROBINS-I tools. *Results*: A total of 24 studies were included: seven on PRP and 17 on PRF. Reported complete mucosal healing rates ranged from 33% to 100% for PRP and from 36% to 100% for PRF. Although two randomized controlled trials and one prospective observational study found no statistically significant advantage of PRF over conventional surgical treatments, most studies indicated positive outcomes. Overall, the methodological quality varied, with several studies showing moderate-to-high risk of bias. *Conclusions*: Platelet concentrates can add benefits to traditional MRONJ treatments. The current evidence suggests that integrating these autologous therapies with conventional approaches clinically enhances healing outcomes, supports bone regeneration, and alleviates symptoms, ultimately leading to improved patient care.

## 1. Introduction

In the 19th century, an epidemic of osteonecrosis exclusively affecting the jaws, termed ‘phossy jaw’, emerged, primarily linked to prolonged exposure to yellow phosphorus [1]. In contemporary clinical practice, osteonecrosis of the jaw (ONJ) encompasses a range of etiologies, the most implicated being antiresorptive and antiangiogenic therapies and radiotherapy [2]. Beyond medication-related osteonecrosis of the jaw (MRONJ) and osteoradionecrosis, ONJ can be characterized as traumatic, non-traumatic, and spontaneous [2].

First recognized in 2003 in bisphosphonate users [3] and later linked to other antiresorptive and antiangiogenic therapies, the condition was renamed from BRONJ to MRONJ in 2014. MRONJ is a rare, yet debilitating condition characterized by pain, swelling, purulent discharge, and bone necrosis [4,5,6,7]. Several biological pathways are implicated in the onset and progression of MRONJ, which includes inhibition of bone remodeling, angiogenesis suppression, chronic inflammation, immune dysfunction, and genetic susceptibility [4,7]. In addition, several risk factors have been associated with MRONJ, including the type and dosage of medication, the presence of periodontal disease, a history of dentoalveolar surgery, and inadequate oral hygiene [4,7].

Despite the availability of both conservative and surgical management options, a definitive treatment standard remains lacking [8]. The increased use of potent antiresorptive agents, such as bisphosphonates and denosumab—particularly at higher doses for malignancy-associated skeletal-related events—has been closely linked to the development of MRONJ [9,10]. As a result, several international expert groups now recommend comprehensive pre-treatment dental evaluations and strict oral hygiene protocols prior to initiating antiresorptive therapy [9,10]. In patients with multiple risk factors undergoing major oral surgery, temporary interruption of therapy may be considered, especially in cancer patients, until surgical sites have healed [10]. A multidisciplinary, preventive approach is advised to mitigate MRONJ risk [9,10]. In cases where MRONJ develops, conservative therapy remains first-line, with local debridement or surgical management considered in non-responsive or advanced cases [9,10]. Adjuvant modalities, such as drug holidays, ozone therapy, hyperbaric oxygen, low-dose laser therapy, autologous platelet concentrates, cell therapy, including autologous stem cell therapy combined or not with bone marrow, and teriparatide (in select osteoporosis patients), have also been explored for symptom relief and enhancement of mucosal healing [8,9,11,12].

Platelet concentrates offer cost-effective, autologous biomolecules that support angiogenesis and wound healing [13,14,15,16]. They promote fibroblast proliferation, extracellular matrix formation, and osteoblast differentiation—processes that remain functional in MRONJ [17]. Autologous platelet concentrates (APCs) can be classified into four distinct categories based on their leukocyte content and fibrin structure [18]. First generation is composed by pure platelet-rich plasma (P-PRP) and leukocyte- and platelet-rich plasma (L-PRP) [18,19]. Both are low-density fibrin matrix [19]. In terms of leukocyte content, P-PRP is leukocyte-poor, whereas L-PRP is leukocyte-rich [18,19]. The use of anticoagulants and coagulation activators characterizes the first-generation APC preparations [18,19]. The reliance of first-generation APCs on anticoagulants, some bovine derived, raises concerns regarding potential cross-reactivity [19].

In PRF formulations, platelet activation and fibrin polymerization occurs naturally [18,19]. Thus, both P-PRF and L-PRF have a high-density fibrin network, which extend the release of growth factors for several days [19]. Due to the high-density fibrin matrix present in P-PRF and L-PRF, the release of growth factors is progressive over time [19]. Second-generation APCs differ in terms of leukocyte content, with P-PRF being leukocyte-poor and L-PRF leukocyte-rich [18].

PRP exhibits short-lived bioactivity, which limits long-term regenerative capacity [19]. The absence of a fibrin scaffold further limits its mechanical support for wound healing [18,19]. Nevertheless, PRF still presents challenges, including the lack of rigidity, the necessity for immediate preparation and application [20]. Due to the high-speed centrifugation, one of the issues of PRF is grounded on cell loss and uneven leukocyte distribution [21]. To address that, advanced platelet-rich fibrin (A-PRF) was developed using a modified protocol with low-speed centrifugation [21]. This adjustment led to an enhancement in leukocyte entrapment, fibrin structure, and growth factor release [21].

Following the introduction of A-PRF in 2014, further innovations appeared, including A-PRF+, titanium-prepared PRF (T-PRF), and heat-compressed PRF (H-PRF), each with distinct biological and clinical advantages [19,22,23]. Given the growing interest in APCs application on MRONJ, this systematic review evaluated the effectiveness of autologous platelet concentrates, including PRP and PRF, in the management of MRONJ. Particularly assessing their impact on healing rates, bone regeneration, and symptom resolution when used as adjuncts to conventional treatments.

## 2. Materials and Methods

### 2.1. Database Searching and Screening

This systematic review was conducted in accordance with the Preferred Reporting Items for Systematic Reviews and Meta-Analyses (PRISMA) methodology [24]. Given the nature of the present study design, ethical approval from an institutional review board was not required. The search strategy was specified in advance and registered at PROSPERO under number CRD42024597961.

Initially, an exhaustive search and definition of the descriptors (MeSH terms) was performed by two researchers. Descriptor terms were defined independently and validated by consensus. No filter, nor time limit or other data restrictions were used when searching the electronic bibliographic databases. A systematic search was conducted in 3 databases: PubMed, Scopus, and Web of Science, in October 2024. Detailed search on PubMed: ((“osteonecrosis” [All Fields] OR “osteonecrosis of the jaw” [All Fields] OR “bone necrosis” [All Fields] OR “jaw necrosis” [All Fields] OR “ONJ” [All Fields] OR “BRONJ” [All Fields] OR “MRONJ” [All Fields] OR “ARONJ” [All Fields])) AND ((“LPRF” [All Fields] OR “leucocyte and platelet-rich plasma” [All Fields] OR “PRF” [All Fields] OR “PRP” [All Fields] OR “platelet-rich fibrin” [All Fields] OR “platelet-rich plasma” [All Fields] OR “plasma rich in growth factors” [All Fields] OR “PRGF” [All Fields] OR “ACSP” [All Fields] OR “CSP” [All Fields] OR “platelet lysate” [All Fields] OR “platelet concentrate” [All Fields] OR “autologous platelet” [All Fields] OR “leukocyte rich plasma” [All Fields] OR “PLRP” [All Fields] OR “leukocyte poor plasma” [All Fields] OR “leukocyte rich platelet rich plasma” [All Fields] OR “leukocyte rich platelet rich plasma” [All Fields])); on Scopus: “osteonecrosis” OR “ osteonecrosis of the jaw” OR “bone necrosis” OR “jaw necrosis” OR “ONJ” OR “BRONJ” OR “MRONJ” OR “ARONJ”; AND: “LPRF” OR “leucocyte and platelet-rich plasma” OR “PRF” OR “PRP” OR “platelet-rich fibrin” OR “platelet-rich plasma” OR “plasma rich in growth factors” OR “PRGF” OR “ACSP” OR “CSP” OR “platelet lysate” OR “platelet concentrate” OR “autologous platelet” OR “leukocyte rich plasma” OR “PLRP” OR “leukocyte poor platelet rich plasma” OR “leukocyte rich platelet rich plasma”; and in Web of Science: TS = (“osteonecrosis” OR “osteonecrosis of the jaw” OR “bone necrosis” OR “jaw necrosis” OR “ONJ” OR “BRONJ” OR “MRONJ” OR “ARONJ”); AND: TS = (“L-PRF” OR “leucocyte and platelet-rich plasma” OR “PRF” OR “PRP” OR “platelet-rich fibrin” OR “platelet-rich plasma” OR “plasma rich in growth factors” OR “PRGF” OR “ACSP” OR “CSP” OR “platelet lysate” OR “platelet concentrate” OR “autologous platelet” OR “leukocyte rich plasma” OR “PLRP” OR “leukocyte poor plasma” OR “leukocyte rich platelet rich plasma”).

### 2.2. Eligibility Criteria

The present systematic review included randomized clinical trials (RCTs), controlled clinical trials, cohort studies, case series involving at least 5 patients, and case reports with a minimum of 5 cases. Exclusion criteria comprised animal studies, in vitro studies, review articles, systematic reviews, meta-analyses, editorials, and letters to the editor. Moreover, studies involving participants under 18 years of age were excluded. Studies unrelated to the research question of the present review or that did not define the reported outcomes precisely or did not assess the primary or secondary outcomes of interest of this review and duplicates were disregarded. No exclusion criteria based on date or language were applied.

The detailed information related to the selection criteria according to the PICO elements is represented in Table 1.

### 2.3. Data Selection

After duplicate elimination, both authors reviewed study’s titles and abstracts using Rayyan tool on blind mode. Subsequently, full-text assessment of the remaining studies was performed and analyzed against inclusion and exclusion criteria.

There were 4 studies whose full text was not publicly available, for which a failed attempt to contact the authors was conducted. Discrepancies were resolved through meetings involving both culminating in consensus. Afterwards, consensus articles, including laser therapies, osteoradionecrosis, and solely preventive approaches, were excluded.

### 2.4. Data Extraction

Two authors independently retrieved quantitative data as well as descriptive statistics data from eligible studies, using a standardized data collection form. The data extracted from each study consisted of authors, year of publication, country, study design, sample size, sex, mean age/range age, intervention, primary disease, comorbidities, antiresorptive/antiangiogenic therapy, and route (if applicable), ONJ type, ONJ stage, preoperative measures, surgery measures, PRP protocol preparation, PRF protocol preparation, postoperative measures, main conclusions, and complications.

### 2.5. Quality and Risk of Bias Assessment

Two independent authors systematically evaluated the study methodologies and assessed the risk of bias for randomized clinical trials using the RoB II tool (Version 2) [25]. Non-randomized studies were assessed according to ROBINS I (Version 2) [26]. To visualize the risk-of-bias assessments, robvis tool (Version 1) was employed [27].

## 3. Results

### 3.1. Study Selection and Screening

**Figure 1:**
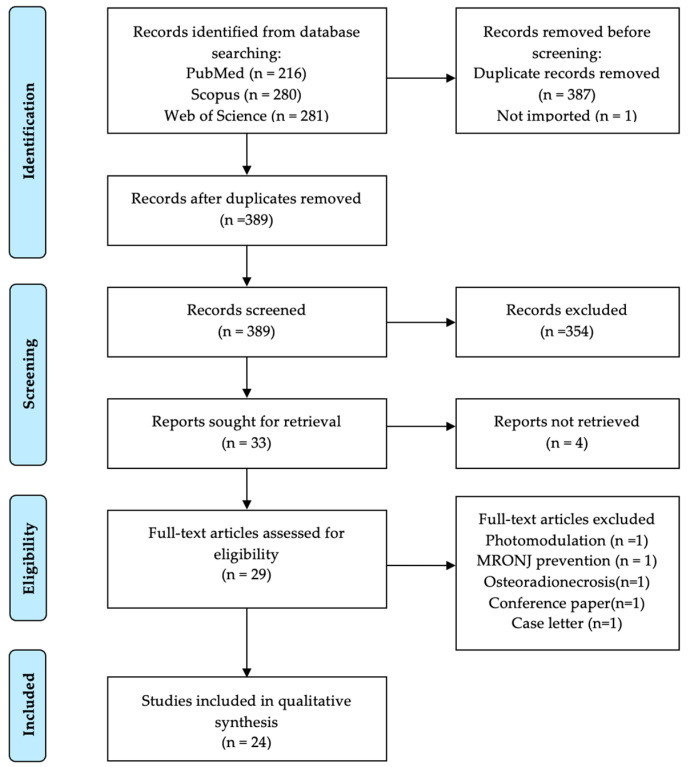
PRISMA flowchart summarizing the selection process.

A total of 777 records were identified through database searching. Following import and removal of duplicates, a total of 389 studies underwent title and abstract screening. Of the 33 articles eligible for full-text assessment, four could not be retrieved due to the unavailability of the full text, despite contacting the respective author. From the 29 articles, comprehensive reasons for exclusion upon full-text assessment were: osteoradionecrosis article (n = 1), MRONJ prevention article (n = 1), photomodulation article (n = 1) conference paper (n = 1), case letter (n = 1). Thus, a total of 24 publications were retained for qualitative synthesis [28,29,30,31,32,33,34,35,36,37,38,39,40,41,42,43,44,45,46,47,48,49,50,51]. Cohen’s kappa was employed to evaluate inter-reviewer concordance, resulting in a coefficient of 0.90 [52]. The study selection process is outlined in Figure 1.

### 3.2. Risk of Bias Assessment

**Figure 2:**
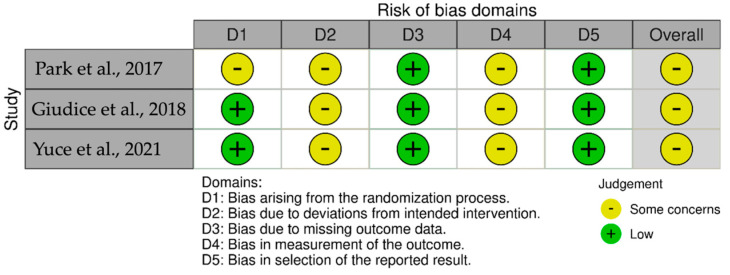
Risk of bias of randomized controlled trials studies according to the RoB-II tool. References: Park et al., 2017 [49], Giudice et al., 2018 [50], Yuce et al., 2021 [51].

**Figure 3:**
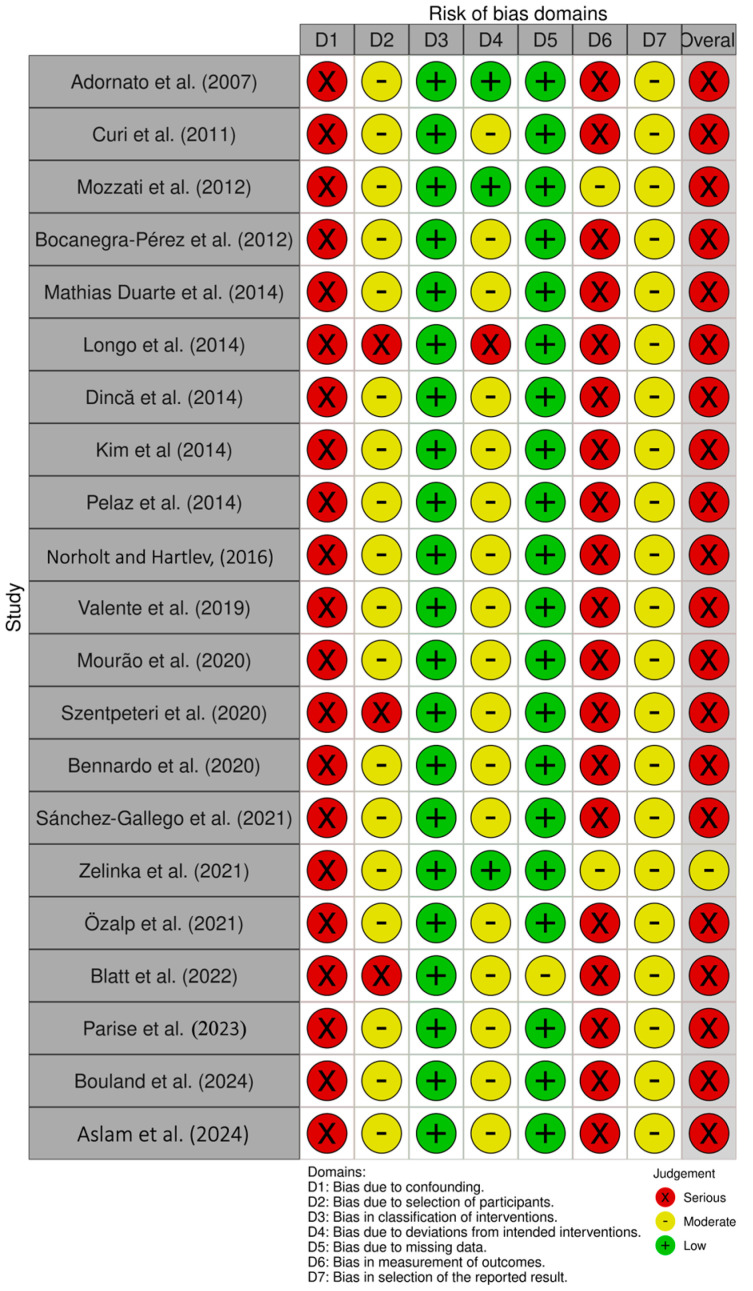
Summary of the risk of bias assessment for non-randomized studies using ROBINS-I. References: Adornato et al., 2007 [28], Curi et al., 2011 [29], Mozzati et al., 2012 [30], Bocanegra-Perez et al., 2012 [31], Mathias Duarte et al., 2014 [32], Longo et al., 2014 [33], Dinca et al., 2014 [35], Kim et al., 2014 [36], Pelaz et al., 2014 [37], Norholt and Hartlev, 2016 [38], Valente et al., 2019 [39], Mourão et al., 2020 [40], Szentpeteri et al., 2020 [41], Bennardo et al., 2020 [42], Sanchez-Gallego et al., 2021 [34], Zelinka et al., 2021 [43], Özalp et al., 2021 [44], Blatt et al., 2022 [45], Parise et al., 2023 [46], Bouland et al., 2024 [47], Aslam et al., 2024 [48].

According to RoB-2, the three RCTs included were found to have an overall moderate risk of bias (Figure 2) [49,50,51]. For the remaining 21 studies included in this review, the ROBINS-I assessments (Figure 3) indicated that one study had a moderate risk of bias [43], while the others were judged to have a high risk of bias [28,29,30,31,32,33,34,35,36,37,38,39,40,41,42,44,45,46,47,48].

### 3.3. Baseline Characteristics of PRP Studies

From all the included studies, year of publication, country, study design, sample size, sex, mean age/range age, primary disease, comorbidities, antiresorptive/antiangiogenic therapy, and route (if applicable), ONJ type, ONJ stage, PRP type, PRP protocol preparation, preoperative measures, surgery measures, postoperative measure, main conclusions, and complications are summarized in Table 2. Of the seven studies investigating the application of PRP for MRONJ/BRONJ management [28,29,30,31,32,33,34], six were case series [29,30,31,32,33,34] and one was a case report [28]. A total of 232 patients were included, of whom 141 received PRP derivatives. The majority were female, with ages ranging from 37 to 85 years.

Regarding antiresorptive therapy, three studies [28,29,30] included only patients treated with intravenous bisphosphonates, while two studies [31,32] involved patients who had received both oral and intravenous bisphosphonates. One study included patients treated with oral bisphosphonates and denosumab [34]. Another study reported the use of bisphosphonates in the study population but did not specify the route of administration [33].

The primary indication for antiresorptive therapy across the studies was malignancy [28,29,30,31,32,33,34]. Notably, no study exclusively included patients with osteoporosis. In four articles, oncologic conditions were the sole underlying disease reported in the study populations [28,29,30,33]. Beyond cancer, osteoporotic patients were reported in three studies [31,32,34]. Patients with other primary conditions were also included in one study [34].

Among the 232 patients included, several comorbidities, habits, and medication use were reported across the studies. These included chemotherapy [28,29,30,31], steroids [30,34], corticosteroids [28,29,31], diabetes mellitus [31,32,34], hypertension [32,34], active smoking [28,30,34], and history of smoking [31]. Only one study did not report any patient-related risk factors [33].

Three studies exclusively included patients with stage II lesions [30,31,32]. Stage I, II, and III MRONJ were presented in two studies [29,34]. One study included patients with lesions across all four stages, including stage 0, which refers to a non-exposed variant characterized by symptoms, clinical signs, or radiologic findings without visible bone exposure [33]. In contrast, one study did not report the lesion stage of the patients [28].

PRP preparations methods were reported in five studies [28,29,30,31,33], with four of them [28,29,30,31] utilizing commercial systems: CS6C [28], SmartPReP [29], PRGF system [30], and the APC-20 Procedure Pack [31]. Centrifugation parameters, the use of activating agents, and the form of application varied across the studies, reflecting a lack of standardization in PRP preparation and delivery protocols [28,29,30,31,33]. APC preparations were not provided in two case series [32,34].

Preoperative protocols were reported in four studies [28,29,30,33] all of which included antibiotic therapy. Specific antibiotics used were amoxicillin [30] and ciprofloxacin [33], while two studies did not specify the antibiotic agent [28,29]. Chlorhexidine was administered in three studies: two as a mouth rinse [28,33] and one as irrigation [29]. Minor bone debridement was performed in two studies [28,29], and one study included a professional oral hygiene session prior to treatment [30]. Preoperative protocols were not reported in three studies [31,32,34]. Regarding intervention, all included studies employed PRP derivatives as an adjunct to surgical treatment [28,29,30,31,32,33,34]. Two studies included patients groups who received either surgical treatment alone or conservative therapy without adjunctive PRP application [32,33].

Postoperative protocols were reported in five studies, all of which included antibiotic therapy [28,29,30,31,33]. Amoxicillin was administered in two studies [30,31], with clavulanic acid co-administration specified in one of them [31]. The remaining studies employed clindamycin [28,29] and ciprofloxacilin [33] as the antibiotic of choice. Chlorhexidine was incorporated into the postoperative regimen in three studies [28,31,33]. Additionally, one study incorporated a professional oral hygiene session as part of postoperative care [31]. No information on postoperative protocols was provided in two studies [32,34].

Most studies reported high healing rates following PRP treatment, ranging from 80% to 100% [28,29,30,31,33,34]. An exception was observed in one study [32], where only two out of six patients (33.3%) achieved complete healing. No serious adverse events were reported as being directly attributable to PRP use. Reported complications were generally mild and varied in incidence, ranging from 0% to approximately 20% (Table 2).

### 3.4. Baseline Characteristics of PRF Studies

Seventeen studies assessing the use of PRF and its variants (L-PRF, A-PRF, i-PRF, CGF) as adjuncts to surgical treatment for MRONJ were included [35,36,37,38,39,40,41,42,43,44,45,46,47,48,49,50,51]. Year of publication, country, study design, sample size, mean age/range age, sex, primary disease, comorbidities, antiresorptive/antiangiogenic therapy, and route (if applicable), ONJ type, ONJ stage, PRF type, PRF protocol preparation, preoperative measures, surgery measures, postoperative measure, main conclusions, and complications are presented in Table 3. Three studies were RCTs [49,50,51].

A total of 489 patients were included across the studies, of whom 327 had ONJ and received PRF derivatives. Most patients were female, with ages ranging from 30 to 97 years. Among those treated with PRF, 174 patients were receiving antiresorptive therapy for oncologic conditions, while 151 were being treated for osteoporosis [36,38,39,41,42,43,44,48,49,50]. Four studies applying PRF included patients exclusively with oncologic diseases as primary disease [35,45,46,47], while three articles included only osteoporotic patients [37,40,51], with one study specifically distinguishing between primary osteoporosis and corticosteroid-induced osteoporosis [37]. The remaining studies included mixed populations comprising both oncologic and osteoporotic patients [36,38,39,41,42,43,44,48,49,50].

All included studies involved patients undergoing bisphosphonate therapy, while a few studies also reported a limited number of patients receiving denosumab [38,39,43,44,45,47,48,50]. Two studies exclusively involved patients on intravenous bisphosphonates [35,46], while three studies comprised only individuals on oral bisphosphonates [37,40,51]. Three other studies reported participants treated with either oral or intravenous bisphosphonates [36,41,49]. Among the included studies, two exclusively enrolled patients with stage II MRONJ [35,40], while two others focused solely on patients with stage III disease [37,42]. Six studies included patients at stages II and III [38,41,44,47,50,51], while four studies encompassed stages I to III [36,45,48,49]. Two studies extended inclusion to stage 0 disease [39,43]. One study reported the inclusion of patients across multiple MRONJ stages but did not provide a detailed breakdown of the distribution by stage [46].

Regarding comorbidities, diabetes was the most reported among the PRF [36,39,40,43,45,47,49,51], followed by smoking [39,43,45,48] and hypertension [39,40,47,51]. Chemotherapy [36,41,43], steroids use [36,48,49], and corticoid [37,39,43] administration were each reported in three papers. Rheumatoid disease [37,45] and renal failure [36,47] were mentioned in two studies each. Six studies did not provide data on comorbidities in their study populations [35,38,42,44,46,50].

Antibiotic therapy was the most consistently reported adjuvant in presurgical interventions [36,38,39,40,41,42,43,44,45,46,48,49,50,51]. Other measures included the use of analgesics [36,49], chlorohexidine [36,42,46,48,49,50], antibacterial mouth rinse [36], professional dental hygiene session [36,42,50] and professional dental prophylaxis [49]. Blood analysis [35] and dental examination [51] were also reported. Additional supportive strategies involved tube feeding [45] and oral hygiene improvement [48]. Presurgical protocols were not reported in two studies [37,47]. Postoperative included antibiotic therapy [35,36,37,38,40,41,42,43,44,45,46,47,49,50,51], dietary modifications [38,40,46,51], analgesics [46,47], antibacterial rinse [36,49], sterile saline irrigation [44] and feeding tubes [45]. Prosthesis use was discouraged in two studies [38,50]. Chlorhexidine mouth rinses [38], irrigation [51], and topical [40] were prescribed after surgery. Postoperative protocols were not reported in one study [48].

The used protocols employed different types of PRF, including PRF [38,40,45], L-PRF [36,39,43,44,46,47,48,49], A-PRF [35,41,50], i-PRF [42], CGF [51] and PRGF [37]. Sixteen of the seventeen included studies provided information on the preparation protocols for PRF [35,36,37,38,40,41,42,43,44,45,46,47,48,49,50,51] and varied among centrifuge used, rotation speeds (rpm), and duration. Centrifuges employed included the IntraSpin EBA 200 (Hettichlab, Tuttlingen-BW, Germany) [40,47], L-PRF centrifuge [38], Process for PRF [50], Daiki DT4000 [46], PRF Duo Centrifuge System [41,45], and EBA 20 [43]. Reported centrifugation speeds and durations were 3200 rpm for 10 min [43], 3000 rpm for 10 min [36,49], 3000 rpm for 8 min [41], 2700 rpm for 12–18 min [48], 2700 rpm for 12 min [40,44,46,47], 1300 rpm for 14 min [35,38], 1300 rpm for 8 min [50], 1200 rpm for 8 min [45], and 700 rpm for 3 min [42]. Additionally, one study used an automated system (Vivostat^®^ System) for PRGF preparation [37]. For CGF preparation, a CGF-specific program with variable speeds and sequential phases using Medifuge System (Silfradent srl, Italy) was employed [51]. One study combined L-PRF with cell therapy, specifically autologous adipose tissue-derived stromal vascular fraction (AT-SVF) [47].

Included studies reported healing rates ranging from 36.9% to 100% [35,36,37,38,39,40,41,42,43,44,45,46,47,48,49,50,51]. Excluding two studies [45,49], PRF healing rates ranged from 69.2% to 100%. Most studies stated favorable outcomes with the addition of PRF, reporting higher healing rates, faster mucosal closure, and reduced recurrence compared to surgery alone.

Three studies did not demonstrate a statistically significant long-term advantage of PRF in the surgical management of MRONJ [45,50,51]. One of these studies reported no significant improvement in wound healing (*p* = 0.302), downstaging (*p* = 0.9), pain reduction (*p* = 0.169), or quality of life (*p* = 0.9) [45]. Another study reported only short-term improvements [50]. Additionally, a randomized study evaluating CGF showed improved clinical outcomes, though these did not reach statistical significance [51]. Overall, the current evidence supports PRF as a beneficial adjunct in MRONJ surgical management (Table 3).

## 4. Discussion

The present systematic review synthesizes the current evidence regarding the use of PRP and PRF derivatives as adjuncts in the surgical management of MRONJ. This complex condition is rare yet a debilitating complication of bisphosphonate and antiangiogenic therapies, with higher prevalence among oncologic patients receiving higher-doses, and intravenous regimens [53,54]. The combination with chemotherapy protocols and corticosteroids poses an additional risk for its development + 53 [55]. MRONJ pathophysiology is multifactorial involving several pathways, including bone suppression via the mevalonate pathway, impaired angiogenesis, soft-tissue toxicity, oxidative stress, immune dysregulation, and genetic factors affecting bone homeostasis [56]. Surgical intervention remains the standard treatment with the indication based on the stage of the pathology, the patient’s clinical condition, and individual needs [57]. To achieve surgical success, tetracycline fluorescence–guided resection can be used to distinguish viable from necrotic bone under UV light, oriented CT–bone scan navigation can be employed for precise mapping and targeting of diseased areas [58,59]. Patients’ comorbidities, such as diabetes, ongoing chemotherapy, and immunosuppression, have been reported as key factors affecting healing [60,61]. To improve the success rates of surgical management, APCs have emerged as a promising minimal complementary procedure.

It was demonstrated that adjuvant PRP and its derivates in clinical practice, may enhance soft-tissue closure and reduce relapse following surgical management of MRONJ. Several PRP studies have reported 80–100% mucosal and bony healing within 6–12 months [28,29,30,31,33,34]. One study reported a significantly higher cure rate of 94% when PRP was added to surgical resection, compared to 53% in the control group [33]. Another study achieved an 81% recurrence-free rate over a four years follow-up period ranging from 2 to 52 months, supporting the biological plausibility that platelet-derived growth factors promote re-epithelialization and bone remodeling following the excision of necrotic tissue [34], improving clinical outcomes. However, outcomes have not been consistently favorable. In one study, only two out of six patients (33.3%) treated with PRP achieved complete healing [32]. This can be related to the severity of the lesions since none of the patients in non-PRP group healed. Therefore, careful clinical assessment is essential to identify patients most likely to benefit from PRP therapy, recognizing that its efficacy correlates closely with lesion severity and proper indication, to maximize therapeutic gain and avoid unnecessary overtreatment.

Across the reviewed studies on PRF, most patients experienced complete healing of MRONJ lesions. Notably, some studies have demonstrated statistically significant benefits of PRF over surgery alone. For instance, one cohort study reported significantly better outcomes in the PRF group compared to the control group, including wound healing, stage improvement, and reduced relapse rate. Specifically, 82.14% of patients treated with PRF achieved wound healing within four weeks, compared to 58.46% in the non-PRF group [41]. In another study, 100% mucosal coverage was observed in the PRF arm versus only 45.5% in control group [48]. In a South Korean randomized controlled trial, the addition of BMP-2 to PRF significantly improved MRONJ healing outcomes compared to PRF alone, indicating that the additional use of BMP may enhance treatment efficacy [49]. The presence of bacterial colonies in biopsy specimens was identified as a significant negative prognostic factor for healing [49]. One case series found an overall success rate of 85%, which increased to 94% when complete bone removal was performed [43], suggesting that lesion size may be an important factor influencing healing outcomes in MRONJ management. Additionally, one study showed that PRF addition not only promoted mucosal integrity, but also reduced pain and infection within the first month postoperatively and the need for re-interventions [50]. Therefore, given the biological properties of PRF and its derivates, and its potential to enhance surgical outcomes in challenging scenarios such as MRONJ, their application may represent a valuable adjunct to improve success rates. The clinical application of PRF suggests it as a valuable adjunct in high-risk or refractory cases to boost success and minimize complications.

However, the long-term impact of PRF on successful outcomes is inconsistent. One RCT found higher 6-month healing rates with concentrated growth factors (CGFs) compared to the control group, but the difference was not statistically significant [51]. Another study found a significant improvement in mucosal integrity with PRF at 1 month, but that was lost after 6 months [50]. This finding suggests that PRF may primarily accelerate the initial phases of wound healing, but its relative advantage diminishes over time. A Germany study reported no significant clinical improvement in wound healing, disease downstaging, pain reduction, or quality of life [45]. Furthermore, it was highlighted that multiple surgical interventions may be necessary to achieve complete healing, underscoring the importance of an optimal surgical technique and complementary protocols [39]. It is likely that APCs act primarily during the early healing phase, with their effects diminishing over time as they are gradually resorbed. While enhanced initial healing may delay recurrence, many other factors, unrelated to APC use, can contribute to recurrence in the subsequent months.

Among the reasons for clinical failure in the treatments, patient-related factors, including the primary indication for antiresorptive or antiangiogenic therapy (malignancy versus osteoporosis), comorbid conditions (e.g., active smoking, diabetes mellitus), and MRONJ stage, significantly modulate healing outcomes [34]. In particular, treatment failures are more common among patients with advanced lesions: one study reported higher failure rates in those with stage II disease [32], another study similarly found that delayed or absent healing correlated with higher-stage BRONJ [36]. A prospective case series reported recurrence in a patient receiving high-dose bisphosphonates who presented with stage III lesion [38]. Moreover, incomplete removal of necrotic bone has also been linked to relapse, predominantly in cases where necrotic bone was not fully debrided [43]. Collectively, these data highlight that both systemic factors (e.g., comorbidities, treatment indication) and local lesion characteristics (e.g., MRONJ stage, completeness of debridement) can influence treatment outcomes. Additionally, the success of surgical procedures, with or without APC therapies, is also influenced by preoperative and postoperative care protocols.

The clinical efficacy of APCs in managing MRONJ remains a contested topic, largely owing to significant variability in preparation protocols and centrifugation parameters [19,62]. PRP studies illustrate this inconsistency: among seven investigations, only four specified commercial systems (CS6C [28], SmartPReP [29], PRGF System [30], and the APC-20 Procedure Pack [31]), and just two detailed centrifugation settings—1800 rpm for 8–10 min [30,33]. Without uniform reporting, it is difficult to draw definitive conclusions about PRP’s role in MRONJ treatment. Nevertheless, the observed heterogeneity in clinical responses underscores the importance of standardizing PRP preparation to determine its true efficacy.

For clinical application, L-PRF emerges more consistently across studies, both in terms of protocol and outcomes. The majority of L-PRF investigations employed centrifugation speeds between 2700 and 3200 rpm for 10–18 min, resulting in mucosal healing rates ranging from 36% to 100% [36,43,44,46,47,48,49]. One randomized trial further demonstrated that combining L-PRF with recombinant human BMP-2 significantly improved healing compared to L-PRF alone [49], suggesting a potential synergistic effect between growth factor supplementation and platelet concentrates. Another study paired L-PRF with AT-SVF, achieving 90% mucosal healing within one month [47]. Collectively, these findings suggest that L-PRF, when prepared under standardized high-speed protocols, offers robust short- and medium-term healing benefits. However, longer-term follow-up data remain limited.

Standard PRF protocols yield more mixed results, reflecting the impact of centrifugation speed on clinical outcomes [38,40,45]. One trial utilizing high-speed centrifugation (2700 rpm for 12 min) reported 100% healing within 2 weeks [40], whereas two studies using lower-speed protocols (1200–1300 rpm for 8–14 min) achieved divergent outcomes: one documented 93% complete mucosal healing without residual symptoms [38], while the other observed no statistically significant improvements in wound healing at six months [45]. These discrepancies highlight how modest alterations in preparation can substantially influence PRF’s clinical performance, and they emphasize the need for head-to-head comparisons of high- versus low-speed PRF protocols.

A-PRF has been explored in only a few investigations, yet initial results are promising [35,41,50]. A cohort study demonstrated that A-PRF achieved 82.14% wound closure at four weeks, along with complete stage improvement [41]. Across two studies employing a centrifugation speed of 1300 rpm for 8–14 min, A-PRF achieved 100% mucosal healing within 30 days in one case series [35] and a randomized controlled study further demonstrated that A-PRF significantly enhanced mucosal integrity and reduced pain at one month compared to controls (87.5% vs. 60.9%), although these differences were no longer significant at six months and one year [50]. This pattern suggests that A-PRF may accelerate early healing but may not confer sustained long-term advantages, calling for further investigation into maintenance protocols and adjunctive therapies.

Finally, i-PRF, prepared using a low-speed centrifugation protocol (700 rpm for 3 min), achieved 75% healing of sinus tract and bone lesion at four weeks [42]. One study using CGF reported a 78.6% healed at six months [51]. Additionally, plasma rich in growth factors (PRGF), obtained using an automated program, resulted in 100% healing [37]. Given the variability in preparation protocols, characteristics of patients and study design, definitive clinical conclusions about the comparative effectiveness of APCs cannot be drawn.

Overall, platelet-derived products can add enhancement in healing for MRONJ lesions by providing a concentrated source of growth factors that promote angiogenesis and tissue regeneration. In summary, L-PRF protocols consistently yield high early healing rates, while PRF outcomes appear highly sensitive to centrifugation speed. A-PRF shows promise for rapid initial recovery, though long-term benefits remain unclear. The diversity in APCs types and preparation techniques complicates direct comparisons and data interpretation.

Despite encouraging outcomes, the current evidence has several limitations. Most studies are limited by small sample sizes and heterogeneity in design, including variations in surgical techniques, follow-up durations, and, in some cases, the absence of clear control groups or standardized outcome measures. Another limitation is the moderate-to-high risk of bias observed across included studies. Furthermore, publication bias cannot be excluded: the predominance of small studies reporting positive outcomes likely reflects selective reporting, since studies with poorer or null results are often not published. Consequently, the true effectiveness may be somewhat lower than that suggested by the existing literature. Nonetheless, the broad geographic and clinical diversity of the studies, along with consistently low postoperative complication rates, support the safety profile of platelet concentrates as an adjunct to surgery. Given the promising yet inconsistent results, future research should prioritize well-powered, prospective randomized controlled trials employing standardized protocols for APC preparation, lesion classification, and outcome assessment.

## 5. Conclusions

Platelet concentrates can offer potential benefits when integrated with conventional treatments for MRONJ. Current evidence indicates that combining these autologous therapies with standard approaches can enhance healing, promote bone regeneration, and alleviate symptoms, thereby improving overall patient outcomes, depending on the clinical case. Both PRP and PRF have demonstrated effectiveness as adjunctive treatments in selected MRONJ/BRONJ cases, contributing to improved healing and reduced recurrence rates. However, the considerable variability in study designs and treatment protocols warrants cautious interpretation. This underscores the need for standardized methodologies and well-designed clinical trials to definitively establish the therapeutic value of PRP and PRF.

## Figures and Tables

**Table 1:** PICO question definition.

Criteria	Determinants
Population	Patients with osteonecrosis of the jaw
Intervention	Platelet-rich plasma (PRP) protocols
Comparator	Platelet-rich fibrin (PRF) protocols
Outcome	Effectiveness in treating osteonecrosis of the jaw (e.g., healing rates, symptom relief, bone regeneration)
Study Design	Randomized controlled trials, case-controlled trials, cohort studies (prospective and or retrospective), case series (≥5 cases), case reports (≥5 cases)

**Table 2:** Summary of the main characteristics of PRP studies included.

Study ID/Country /Design	Population	APC Type/APC Preparation	Preoperative/Surgery/Postoperative	Results	Complications
[28]/USA/ Case Report	12 oncologic BON on chemotherapy and corticoids, smoking history, 8 females, (43–83 years) IV BPs	PDGFs/CS6C, Vulcun technologies (automated centrifuge) for 11 min. Thrombin and CaCl_2_ mixed and added to PRP to form gel.	Minor bone debridement + CHX oral rinse + antibiotics/bone resection + PRP topical gel + resorbable collagen membrane with PRP/clindamycin 300 mg + CHX rinse	10/12 (83%) complete mucosal and bone healing after 6 months	2 partial healing
[29]/Brazil/ Case Series	25 oncologic, BRONJ, most on chemotherapy and corticoids, 20 females, 60.7 years (42–85) IV BPs	PRP/SmartPReP system	Minor bone debridement + CHX oral irrigation + antibiotics/IV clindamycin 600 mg + marginal resection (teeth within 1 cm were extracted) + PRP topical/clindamycin 600 mg	20/25 (80%) complete mucosal healing and no exposed bone after 12 months.	2 infections during conservative treatment. 1 recurrence
[30]/Italy /Case Series	32 oncologic, BRONJ (IIB) chemotherapy, steroids, smokers, 22 females, 69.7 years (44–83), IV BPs	PRGF/ PRGF System at 1800 rpm for 8 min. PRGF activated with 10% calcium chloride to form a gel	Hygiene session + amoxicillin/marginal resection (teeth within 3 mm removed) + osteoplasty + bone oxygenation + PRGF membrane/amoxicillin	32/32 (100%) healed No recurrence (48–50 months)	1 paresthesia (resolved), 4 with pain for 10 days
[31]/Spain/Case Series	6 oncologic and 2 osteoporotic, BRONJ (stage II and < 3 cm), DM, corticosteroids, chemotherapy, former smokers, 6 females, 66 years (55–77), oral and IV BPs	L-PRP/ APC-20 Procedure Pack. PRP activated by mixing with thrombin	N.R./surgical debridement + L-PRP/amoxicillin-clavulanic acid 875 mg + CHX mouthwash + oral hygiene	Lesion healed in 2–4 weeks; asymptomatic, no exposed bone for 12–26 months	N.R.
[32]/Brazil/Retrospective case series	10 oncologic and 3 osteoporotic, BRONJ (stage II), DM, HTA,12 females, 67.3 years (48–84), oral and IV BPs	PRP/N.R.	N.R./ Conservative (*n* = 3) clindamycin 300 mg + CHX irrigation Surgical resection(*n* = 10) - with PRP (*n* = 6) - without PRP (*n* = 4)/ N.R.	2/6 (33.3%) healed with PRP 0/4 healed without PRP 2/3 healed with conservative.	3 patients remained in stage II (1 from each group) 5 regressed to stage I; 2 remained
[33]/Italy/Retrospective case series	72 oncologic, BRONJ, 60 females, 59 years (37–81), BPs	PRP/180 rpm for 10′ min, then at 1800 rpm for 10′ min. Calcium gluconate added to PPP for thrombin formation. 1800 g for 10–15 min. Thrombin ionized Ca added to PRP to form gel.	Oral ciprofloxacin 500 mg + CHX rinse/curettage with or without necrotic bone excision with PRP(*n* = 34) or without PRP (*n* = 15)/ Oral ciprofloxacin 500 mg + CHX rinse	23/72 (32%) healed with conservative treatment 32/34(94%) healed with surgery + PRP 8/15 (53%) healed with surgery alone	2/34 (6%) partial healing with surgery and PRP 7/15 (47%) partial healing with surgery alone
[34]/Spain/Retrospective case series	34 osteoporotic and 29 oncologic, 7 others, MRONJ, steroids, DM, HTA, smokers, 58 females, most 50–70 years, oral BPs and denosumab	PRP gel/N.R.	N.R./bone resection (teeth extracted if near) + PRP gel/N.R.	57 (81.4%) had not recurred for 2–52 months.	13/70 (18.6%) experienced recurrence Smoking independent risk factor for recurrence.

Legend: ON—Bisphosphonate Osteonecrosis, BPs—Bisphosphonates, BRONJ—Bisphosphonate-Related Osteonecrosis of the Jaw, CHX—Chlorhexidine, HTA—hypertension, DM—Diabetes mellitus, IV—Intravenous, L-PRP—Leukocyte and Platelet-Rich Plasma, mg—milligram, min—minutes, mm—millimeters, MRONJ—Medication-Related Osteonecrosis of the Jaw, N.R.—Not Reported, PDGF—Platelet-derived Growth Factor, PRGF—Plasma Rich in Growth Factors, PRP—Platelet-rich plasma, rpm—rotations per minutes.

**Table 3:** Summary of the main characteristics of PRF studies included.

Study ID/Country/ Design	Population	APC Type/APC Preparation	Preoperative/ Procedure/Postoperative	Results	Complication
[35]/Romania/Retrospective Case Series	10 oncologic, BRONJ (stage II), 6 females, 59 ± 15 years (30–79), IV BPs	A-PRF/1300 rpm, 14 min, no anticoagulant.	Blood analyses/superficial sequestrectomy + PRF clots/clindamycin 0.9 g	10/10 (100%) complete mucosal healing and no exposed bone at 30 days	N.R.
[36]/South Korea/Prospective feasibility study	32 osteoporotic and 2 oncologic, BRONJ, chemotherapy, steroids, DM, obesity, RF, 34 females, 71 ± 13 years IV and oral BPs	L-PRF/3000 rpm, 10 min, no anticoagulant	IV cephalosporin 1 g + analgesics + CHX irrigation + antibacterial rinse + professional hygiene session/necrotic bone removal, sequestrectomy, ostectomy+ antibiotics irrigation + L-PRF/antibacterial rinse + systemic antibiotics	26/34 (77%) no exposed or necrotic bone at the site, full coverage by mucosa, and no pain at 1 month Association between the response to treatment and the stage of BRONJ (*p* = 0.002) No association between the response to treatment and sCTX concentrations, actinomycosis or site of lesion.	6/34 (18%) expose or necrotic bone at 1 month but resolved at 4 months (delayed). 2/34 (6%) no healing
[37]/Spain/Non-randomized comparative pilot study	PRF 3 osteoporosis and 2 osteoporosis corticoids related, 72.8 years (60–87) Teriparatide 2 osteoporosis and 2 osteoporosis corticoids related, 73.5 years (64–84) BRONJ (stage III), RD, corticoids, MTX, 9 females Oral BPs	PRGF/Vivostat PRF^®^ System (automated)	N.R./ PRF sequestrectomy + Curettage + PRF Teriparatide teriparatide 20 µg/day, 4–10 months/ PRF amoxicillin-clavulanic acid 4 g/day	PRF 5/5 (100%) complete healing Teriparatide 1/4 (25%) complete healing	PRF Lip anesthesia (1), oro-antral communication (1)—resolved Teriparatide Bone exposure (3) (1 symptomatic)
[38]/Denmark/Prospective case series	8 oncologic and 7 osteoporotic, BRONJ (stage II and III), 68.5 years (54–83), 11 females; BPs and denosumab	PRF membrane/1300 rpm, 14 min (L-PRF centrifuge). Fibrin clots pressed to form a membrane	PO 2 MIU penicillin (if allergy: clindamycin) + metronidazole 1 g/IV antibiotic + bone resection + PRF membranes/metronidazole 0.5 g + 1 MIU penicillin (if allergy: clindamycin) + prothesis avoidance + CHX rinse + soft diet	14/15 (93%) complete mucosal healing and no symptoms (follow-up: 7–20 months)	1/15 (7%) recurrence with bone exposure (high dose, bilateral mandibular involvement, stage III, died of cancer after 14 months)
[39]/Switzerland/Retrospective clinical study	8 osteoporotic and 7 oncologic, MRONJ, HTA, HCL, smoking, DM, AF, previous MI, corticoids, 9 females,69 years (56–71); IV, oral BPs Denosumab SC	L-PRF/N.R.	Antibiotic Therapy (15): amoxicillin 2–3 g clindamycin 900–1200 mg ciprofloxacin 500 mg /sequestrectomy + PRF + antibiotics (1) bone debridement + PRF + antibiotics (13) Number of surgeries: 1 (3), 2 (8), 3 (3) /antibiotic cycle repetition	11/15 (73.3%) treated with L-PRF achieved complete healing (mean period: 42.2 months)	8/11 reintervened (>1) for healing 4/15(26.7%) relapsed
[40]/Brazil/Case Series	11 osteoporotic, MRONJ (stage II), HTA, DM, 9 females, 67.7 ± 14.6 years (38–84), oral BPs	PRF/2700 rpm, 12 min without anticoagulants (Intra-Spin EBA 200). PRF clot pressed with PRF-Box1 (Intra-Lock System, Miami-FL, USA)	Amoxicillin-clavulanic acid 875/125 mg /necrotic bone removal + debridement + PRF membranes/oral amoxicillin-clavulanic acid 875/125 mg + soft diet + topical CHX	11/11 (100%) complete healing at 2 weeks	N.R.
[41]/Hungary/Retrospective Cohort Study	PRF 25 oncology and 3 osteoporosis, 68.42 years Control 61 oncology and 12 osteoporosis, 63.97 years. MRONJ (stage II and III), chemotherapy, antihormonal therapy, 74 females oral and IV BPs	A-PRF membrane/ 3000 rpm, 8 min. without anticoagulants (PRF Duo Centrifuge System, Process for PRF), A-PRF layer processed into a membrane	Oral amoxicillin-clavulanic acid 875 mg/125 mg (if allergy: clindamycin 300 mg)/necrotic bone debridement ± PRF membranes/ same regimen	Control: 38/73 (58.46%) wound healing at 4 weeks 54/70 (77.14%) stage improvement PRF: 23/28 (82.14%) wound healing at 4 weeks 28/28 (100%) stage improvement Wound healing (*p* = 0.022), stage improvement (*p* = 0.005), and reduced relapse rate (*p* < 0.001) significantly superior in PRF group	Control: 25/38 (65.78%) relapsed PRF: 5/23 (21.74%) relapsed
[42]/Italy/Case report	5 osteoporotic and 3 oncologic, MRONJ with facial sinus tracts (stage III), 6 females (65–82 years), BPs	i-PRF/700 rpm for 3 min without additives (i-PRF, process for PRF)	Oral hygiene session + CHX mouthwash + amoxicillin 1 g (if allergy: clindamycin 600 mg) + metronidazole 250 mg/bone debridement + Sinus tract management (1 mL of PRF near fistula, weekly for 1 month)/same antibiotic regimen	6/8 (75%) healing of sinus tract and bone lesion at 4 weeks pain relief 8/8 (100%) within 2 days	1 (12.5%) persistent bone lesion and fistula 1 (12.5%) incomplete mucosal cover at 4 weeks
[43]/Czech Republic/Prospective case series	34 oncologic and 6 osteoporotic, MRONJ, DM, corticosteroids, chemotherapy, smoking, 24 females, 69 years (37–85), BP or/and denosumab	L-PRF/ 3200 rpm, 10 min. without anticoagulants (EBA 20).	Amoxicillin + clavulanic acid 875 mg/125 mg (if allergy clindamycin 300 mg)/sequestromy + debridement necrotic bone (except 4 cases) + PRF clots/antibiotics	34/40 (85%) complete healing at 12 months Significant association between size of necrotic bone and treatment response (*p* = 0.014)	6/40 (15%) recurred 4/6 incomplete necrotic bone removal 1/6 extraoral fistula 1/6 multiple intraoral fistulas
[44]/Turkey/Clinical study	10 oncologic and 3 osteoporotic, MRONJ (stage II and III), 7 females, 72.4 ± 10.61 years (54–84); IV, oral BPs and denosumab	L-PRF/2700 rpm, 12 min. without anticoagulants	Oral Amoxicillin-clavulanate 875/125 mg, (allergy: clindamycin 150 mg)/ Marginal resection + L-PRF (3) sequestrectomy + peripheral ostectomy + L-PRF (9) curettage + L-PRF (1)/ systemic antibiotics + sterile saline irrigation	9/13 (69.2%) complete healing	4/13 (30.8%) incomplete healing 1 oro-antral fistula partially resolved 1 chronic fistula and pain resolved after 3 surgeries 1 persistent exposed bone after 4 surgeries 1 resolved after 2nd intervention
[45]/Germany/Non-Interventional Prospective Observational Study	52 oncologic, MRONJ, DM, RD osteoporosis, smoking, immunomodulatory therapy, 27 females, 71.5 ± 8.6 years, IV BPs or denosumab SC	PRF membrane/1200 rpm, 8 min (duo centrifuge, process for PRF) PRF membrane	Ampicillin/sulbactam 3 g IV, (if allergy: clindamycin 600 mg PO) + tube feeding/ Arm A (*n* = 22): surgical resection Arm B (*n* = 30): surgical resection + PRF/ same regimen	No significant wound healing, downstaging, pain reduction, or quality of life	16 (30.76%) wound dehiscence
[46]/Brazil/Case-control study	20 oncologic, MRONJ, 12 females, 61.9 years (41–91), IV BPs	L-PRF/2700 rpm, 12 min (DT4000, Daiki)	CHX rinse + amoxicillin 500 mg + metronidazole 400 mg/ Group 1 tooth extractions Group 2 tooth extractions + L-PRF Group 3 (MRONJ) surgery+ L-PRF/pasty liquid diet + analgesics + oral amoxicillin 500 mg + metronidazole 400 mg	Group 1 4/7 (57%) Group 2 8/8 (100%) Group 3 4/5 (80%) achieved mucosal healing and symptom resolution	1 in group 3 had recurred 2 in group 1 developed MRONJ Delayed healing (8–12 weeks) in Group 1
[47]/Belgium/Prospective clinical study	9 oncologic, MRONJ (stage II and III), HTA, DM, HF, RF, 6 females, 68 ± 8 years, BPs or denosumab	L-PRF/AT-SVF/2700 rpm, 12 min, no anticoagulants to obtain L-PRF. Lipoaspiration. Enzymatic treatment and centrifugation. SVF pellet harvested + saline solution. AT-SVF injected into L-PRF (Intra-SpinEBA 200)	N.R./ debridement + AT-SVF/L-PRF/analgesics + amoxicillin-clavulanic acid, 875 mg	9/10 (90%) lesions of oral mucosa healed within 1 month. 8/9 imaging showed bone healing at 6 months	2/9 new MRONJ lesions In different locations 1/9 died from cancer after 6 months of follow-up 1/9 imaging showed rejected the bony sequestrum naturally after 12 months
[48]/UK/Retrospective Observational study	Control 8 oncologic, 2 osteoporosis and 1 other, PRF 7 osteoporosis, 3 oncologic and 1 other MRONJ, smoking, steroids, 72 ± 8.08 years (58–87), 20 females oral, IV BPs and denosumab	L-PRF/2700 rpm, 12–18 min, no anticoagulants	Oral hygiene improvement + CHX mouthwash + oral antibiotics (if infection)/ Bone debridement/ sequestrectomy ± L-PRF/N.R.	Control 5/11 (45.5%) complete healing PRF 11/11 (100%) complete healing Statistically significant (*p* = 0.004)	1 further reintervention 2 deceased with exposed bone 3 required additional treatments.
[49]/South Korea/RCT	PRF 22 osteoporosis and 3 bone metastases, 75.24 years (59–97) PRF+BMP2 26 osteoporosis, 4 bone metastasis, 75.2 years (60–85) MRONJ, DM, steroids, 51 females oral, IV BPs	L-PRF/3000 rpm, 10 min.	IV 3rd generation cephalosporin 1 g + Analgesics + CHX irrigation + professional dental prophylaxis/ necrotic bone debridement + antibiotic irrigation + L-PRF ± collagen sponge with rhBMP-2/ antibacterial mouth rinse + antibiotics	PRF 9/25 (36.0%) complete healing at 4 weeks PRF+BMP2 18/30 (60.0%) healing at 4 weeks Statistically significant (*p* = 0.028) Bacterial colonies significant negative factor affecting healing (*p* =0.017)	13/25 (52.0%) delayed healing with PRF 11/30 (36.7%) delayed healing with PRF+BMP2 3/25 (12%) no healing with PRF alone 1/30 no healing with PRF+BMP2
[50]/Italy/RCT	PRF 19 oncologic and 5 osteoporotic, 75.5 ± 5.6 Control 16 oncologic and 7 osteoporotic, 73.9 ± 7.4 years MRONJ (stage II and III), 24 females, Oral, IV BPs and SC denosumab	A-PRF membrane/1300 rpm, 8 min (in a specific centrifuge, process for PRF). PRF Box surgical kit to form membrane	Professional hygiene session + amoxicillin 1 g (allergy: clindamycin 600 mg) + metronidazole 250 mg + CHX mouthwash/ debridement ± A-PRF membrane/ same antibiotic regimen + prothesis avoidance	Mucosal Integrity PRF = 87.5%, Control = 60.9% (*p* < 0.05) at 1 month No significant differences at 6 months or 1 year Reduced pain and fewer postoperative infections in PRF group at 1 month (*p* < 0.05)	Reinterventions 1 month PRF = 3 patients Control = 9 patients 6 months PRF =1 patients Control =2 patients (*p* < 0.05) Signs of fistulas 6 months PRF = 1 patients Control = 1 patients (both received high dose therapy)
[51]/Turkey/RCT	CGF 14 osteoporotic, 73.57 ± 5.1 (65–81) Control 14 osteoporotic, 73.64 ± 5.49 (65–81) MRONJ (stage II and III), DM, HTA, AD, 28 females, 65–81 years Oral BPs	CGF/30 s accelerations, 2700 rpm, 2 min, 2400 rpm, 4 min, 2700 rpm, 4 min, 3000 rpm, 3 min, 36 s deceleration. No anticoagulants	Dental examination + PO amoxicillin-clavulanic acid 2 g/ sequestrectomy + curettage + CGF clots/ Soft diet + CHX irrigation+ antibiotic (if infection)	CGF 11/14 (78.6%) healed at 6 months Control 8/14 (57.1%) healed at 6 months No statistically significant differences (*p* > 0.05)	CGF: 3 bone exposure (1 also infected) Control: 6 bone exposure (3 also infection)

Legend: AD—autoimmune disease, AF—atrial fibrillation, A-PRF—Advanced Platelet-Rich Fibrin, AT-SVF—Adipose-Tissue-derived Stromal Vascular Fraction, BMP-2 (rh)—(recombinant human) Bone Morphogenetic Protein-2, BPs—Bisphosphonates, BRONJ—Bisphosphonate-Related Osteonecrosis of the Jaw, CGF—Concentrated Growth Factor, CHX—Chlorhexidine, DM—Diabetes Mellitus, HCL—Hypercholesterolemia, HF— Heart failure, g—gram, HTA—hypertension, i-PRF—Injectable Platelet-Rich Fibrin, IV—Intravenous, L-PRF—Leukocyte- and Platelet-Rich Fibrin, mg—milligram, min—minutes, MI— Myocardial infarction, MIU—Million International Units, mL—milliliters, MRONJ—Medication-Related Osteonecrosis of the Jaw, MTX— Methotrexate, µg—microgram, N.R.—Not Reported, PO—per os (oral), PRF—Platelet-Rich Fibrin (unspecified subtype), PRGF—Plasma Rich in Growth Factors, RD—Rheumatoid disease, RF—renal failure, rpm—rotations per minutes, RCT—randomized controlled trial (commonly abbreviated RCT), SC—subcutaneous.

## Data Availability

All data generated during this study is available upon reasonable request from the corresponding author.

## Abbreviations

The following abbreviations are used in this manuscript:

AD	Autoimmune disease
AF	Atrial fibrillation
APC	Autologous platelets concentrates
A-PRF	Advanced platelet-rich fibrin
AT-SVF	Adipose-tissue stromal vascular fraction
BMP2	Bone morphogenetic protein 2
BPs	Bisphosphonates
BRONJ	Bisphosphonate-related osteonecrosis of the jaw
CGF	Concentrated growth factor
CHX	Chlorhexidine
CS	Case Series
DM	Diabetes
HCL	Hypercholesterolemia
HF	Heart Failure
HTA	Hypertension
i-PRF	Injectable platelet-rich fibrin
IV	Intravenous
L-PRF	Leukocyte-platelet rich fibrin
L-PRP	Leukocyte- and platelet-rich plasma
MI	Myocardial infarction
Min	Minutes
MIU	Million international units
MRONJ	Medication-related osteonecrosis of the jaw
MTX	Methotrexate
N.R.	Not reported
ONJ	Osteonecrosis of jaw
PDGF	Platelet-derived growth factor
PO	Per os
PPP	Platelet-poor plasma
PRF	Platelet-rich fibrin
PRGF	Plasma rich in growth factors
PRP	Platelet-rich plasma
RCT	Randomized control trial
RD	Rheumatoid disease
RF	Renal Failure
rpm	Rotations per minute
SC	Subcutaneous
SVF	Stromal vascular fraction
